# Device-Related Adverse Events and Modes of Failure in Renal Denervation Systems: Insights From the Food and Drug Administration Manufacturer and User Facility Device Experience (FDA MAUDE) Database Analysis

**DOI:** 10.7759/cureus.96770

**Published:** 2025-11-13

**Authors:** Manasa Jasti, Ashish Kumar, Aravinda Nanjundappa, Ankur Kalra, Timir Paul

**Affiliations:** 1 Department of Cardiovascular Science, Ascension St. Thomas Hospital, University of Tennessee Health Science Center, Nashville, USA; 2 Department of Cardiovascular Medicine, Mayo Clinic, Rochester, USA; 3 Department of Cardiovascular Medicine, Cleveland Clinic, Cleveland, USA; 4 Department of Cardiology, Indiana University School of Medicine, West Lafayette, USA; 5 Department of Cardiology, Krannert Cardiovascular Research Center, Indiana University School of Medicine, Indianapolis, USA; 6 Department of Cardiology, Franciscan Health, Lafayette, USA

**Keywords:** complications, device-related adverse events, endovascular therapy, failure modes, hypertension, renal denervation

## Abstract

Renal denervation (RDN) therapy has emerged as a device-based treatment option for patients with resistant hypertension. There are currently two Food and Drug Administration (FDA) approved RDN systems in the United States: the Paradise^R^ RDN system (Recor Medical, Palo Alto, California) and the Symplicity Spyral^TM^ RDN system (Medtronic, Minneapolis, Minnesota). There are limited data on commonly reported adverse events and failure modes associated with these devices. Our objective is to assess the frequently reported adverse events and modes of failure for RDN therapy with the two FDA-approved devices by analyzing data from the FDA Manufacturer and User Facility Device Experience (MAUDE) database. The MAUDE database was queried for the terms “renal denervation”, “Symplicity Spyral”, and “Paradise renal” for reports received by the FDA to yield a total of 61 results (44, 17, and 0 results, respectively), with the earliest event from November 9, 2016, to the latest on January 5, 2024. After adjusting for duplicates and excluding results unrelated to RDN therapy (n = 38), a total of 23 results pertaining to 13 cases were identified. All the events that resulted from the MAUDE database were Medtronic Symplicity Spyral^TM^-related. The most common complication reported was renal artery perforation or dissection (n = 7), of which five were intraprocedural vessel dissection and two were postprocedurally identified vessel perforation. Among the five intraprocedural vascular dissections, four were secondary to the guide wire. Other complications (n = 3) included intraprocedural bradycardia requiring atropine (n = 1) and postprocedural bleeding at the right groin access site (n = 2). Reported modes of failure for the RDN system were damage of the device component (n = 2) and device entrapment (n = 1). MAUDE database analysis to date has shown that the most common complication with the RDN system is renal artery dissection or perforation. RDN-related complications appear to be rare. These data provide valuable insight to both operators and manufacturers in further optimizing device performance and enhancing clinical outcomes. Further real-world data/clinical trials are needed to assess implications at a larger scale.

## Introduction

Renal denervation (RDN) therapy has emerged as a device-based treatment option for patients with resistant hypertension or who are unable to tolerate long-term medications or have issues with medication adherence. It is a minimally invasive, endovascular, catheter-based procedure via femoral artery access using radiofrequency or ultrasound energy to ablate sympathetic nerves around the renal arteries [1]. There are currently two Food and Drug Administration (FDA)-approved RDN systems in the United States [2]. The Paradise^R^ RDN system (FDA-approved in November 2023; Recor Medical, Palo Alto, California) uses ultrasound energy to denervate the sympathetic renal nerves, while the Symplicity Spyral^TM^ RDN system (FDA-approved in November 2023; Medtronic, Minneapolis, Minnesota) uses radiofrequency energy to disrupt the renal sympathetic system [2]. There are limited data on commonly reported adverse events and failure modes associated with these devices.

This article is a research letter and was previously presented as a moderated abstract presentation at the Transcatheter Cardiovascular Therapeutics 2024 on October 28, 2024.

## Technical report

The objective of the current study was to report adverse events and modes of failure associated with RDN therapy using the two FDA-approved devices by analyzing data from the FDA Manufacturer and User Facility Device Experience (MAUDE) database [3]. The MAUDE database was queried for the terms “renal denervation”, “Paradise renal”, and “Symplicity Spyral” for reports received by the FDA to yield a total of 61 results (44, 17, and 0 results, respectively), with the earliest event from November 9, 2016, to the latest on January 5, 2024. This comprehensive search was performed in April 2024. This analysis included reports pre-dating FDA approval. The authors think these events may be due to device use in clinical trials, investigational device use, or pre-FDA approval clinical use. After adjusting for duplicates and excluding results unrelated to RDN therapy (n = 38), a total of 23 results pertaining to 13 cases were identified. These 13 cases were included in the final analysis.

All the RDN system-related events that resulted from the MAUDE database search were Medtronic Symplicity Spyral^TM^-related. There were no results yielded for the Recor's Paradise system. The most common complication reported was vascular perforation or dissection (n = 7), of which five were intraprocedural vessel dissections and two were postprocedurally identified vessel perforations. Among the five intraprocedural vascular dissections, four were secondary to the guide wire. The remaining case was a right iliac artery dissection occurring after contrast injection via guide catheter, which was deemed as procedure-related but not as device or RDN-therapy-related as per the MAUDE database. The anatomical breakdown of intraprocedural vascular dissections included left renal lower branch artery (n = 1), left renal branch artery (n = 1), main renal artery (n = 1), and undocumented (n = 1). The case involving the main renal artery dissection was complicated by renal infarction requiring surgical intervention. The two postprocedurally identified vessel perforations involved the renal artery.

**Figure 1:**
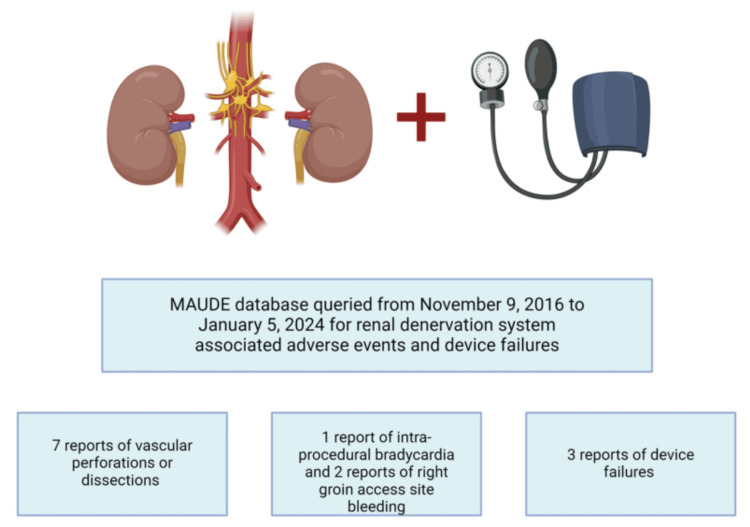
Results of FDA MAUDE database analysis for renal denervation systems Device-related adverse events and modes of failure related to renal denervation systems derived from the analysis of the FDA MAUDE database for events between November 2016 and January 2024. There were seven reports of vascular perforation or dissection, one report of bradycardia, two reports of access site bleeding, and three reports of device failures FDA MAUDE: Food and Drug Administration Manufacturer and User Facility Device Experience Image credit: Created in BioRender. Kumar A. (2025) https://BioRender.com/j37h502

Other complications (n = 3) included intraprocedural bradycardia requiring atropine (n = 1) and postprocedural bleeding at the right groin access site (n = 2), of which one required lidocaine-epinephrine injection infiltration and one was self-resolving with a minor right groin hematoma. Reported modes of failure for the RDN system were damage of the device component (n = 2) and device entrapment (n = 1) (Figure 1).

## Discussion

The analysis of the MAUDE database is a helpful tool for understanding the commonly reported adverse events and failure modes associated with RDN devices. Robust data were not available at the time of analysis, which may be due to RDN being a relatively new therapy in a real-world setting. With limited data and no reports noted for one of the devices, there could be a possibility of limited use, underreporting, or other factors affecting the smaller data on these newly approved devices. This limitation could limit the generalizability of the findings to the real world. Future data, when available, will further increase the power and add to the existing literature. The data reported in the current analysis are similar to those from clinical trials on RDN procedural safety and success [4,5]. The SYMPLICITY HTN-3 trial reported one vascular complication requiring treatment and 99.7% device and procedural success. The study reported no bleeding events. Atropine was used in 2.8% of the patients undergoing RDN therapy [6]. A meta-analysis of 15 randomized controlled trials predominantly reported vascular and bleeding complications, and bradycardia in patients undergoing RDN therapy [7]. Similarly, the current analysis of the MAUDE database to date reported the most common complications to be vascular dissection or perforation with the RDN system. A limitation to note with MAUDE data is its voluntary and passive surveillance nature, which can lead to reporting bias when compared to research trials data. Additionally, a lack of denominator does not give us the true incidence of events.

## Conclusions

RDN therapy has emerged as a device-based treatment option for patients with resistant hypertension. There are limited data on commonly reported adverse events and failure modes associated with these devices. The MAUDE database analysis to date has shown that the most common complication with the RDN system is renal artery dissection or perforation. Other complications included intraprocedural bradycardia and postprocedural bleeding at the access site. Reported failure modes with the RDN system included damage to the device component and device entrapment. RDN-related complications appear to be rare. Given the limited nature of these data, this can be considered hypothesis-generating and guides the need for future research. These early data provides valuable insight to both operators and the manufacturers in further optimizing device performance and enhancing clinical outcomes. Further real-world data/clinical trials are needed to assess implications at a larger scale.
